# Ecological traits interact with landscape context to determine bees’ pesticide risk

**DOI:** 10.1038/s41559-023-01990-5

**Published:** 2023-02-27

**Authors:** Jessica L. Knapp, Charlie C. Nicholson, Ove Jonsson, Joachim R. de Miranda, Maj Rundlöf

**Affiliations:** 1 Department of Biology, Lund University, Lund, Sweden; 2 Department of Aquatic Sciences and Assessment, SLU Centre for Pesticides in the Environment, Swedish University of Agricultural Sciences, Uppsala, Sweden; 3 Department of Ecology, Swedish University of Agricultural Sciences, Uppsala, Sweden; 4 Present Address: Department of Botany, Trinity College Dublin, Dublin, Ireland

**Keywords:** Ecology, Agriculture

## Abstract

Widespread contamination of ecosystems with pesticides threatens non-target organisms. However, the extent to which life-history traits affect pesticide exposure and resulting risk in different landscape contexts remains poorly understood. We address this for bees across an agricultural land-use gradient based on pesticide assays of pollen and nectar collected by *Apis mellifera, Bombus terrestris* and *Osmia bicornis*, representing extensive, intermediate and limited foraging traits. We found that extensive foragers (*A. mellifera*) experienced the highest pesticide risk—additive toxicity-weighted concentrations. However, only intermediate (*B. terrestris*) and limited foragers (*O. bicornis*) responded to landscape context—experiencing lower pesticide risk with less agricultural land. Pesticide risk correlated among bee species and between food sources and was greatest in *A. mellifera*-collected pollen—useful information for future postapproval pesticide monitoring. We provide foraging trait- and landscape-dependent information on the occurrence, concentration and identity of pesticides that bees encounter to estimate pesticide risk, which is necessary for more realistic risk assessment and essential information for tracking policy goals to reduce pesticide risk.

## Abstract (web summary)

Analysing pesticide residues in pollen and nectar collected by three bee species along a land-use gradient, the authors show that extensive foragers like *Apis mellifera* have higher pesticide risk than species that forage at intermediate or limited ranges, irrespective of landscape context.

## Main

Agricultural intensification includes concomitant reductions in seminatural areas and increased reliance on pesticides^1,2^, threatening beneficial insects such as bees that sustain ecosystem function and services^3,4^. Pesticides have received particular attention due to their widespread use yet sometimes detrimental effects on bee individuals^5^, colonies^6,7^, populations^8,9^ and pollination services^10,11^. As pesticide risk (summed toxicity-weighted concentrations) depends on exposure (the degree to which an organism encounters pesticides at a given time and place) it is vital to determine how bee activity patterns intersect with the occurrence, concentration and identity of pesticides^12^.

Pesticide-treated cropland, especially of intensively managed fruit and vegetable crops, can increase the amount and diversity of pesticides in the landscape^13–16^. However, pesticides do not just affect target crops and their pests; they can drift and leach into the surrounding air, soil and water to contaminate non-crop plants^17–21^. Thus, seminatural habitats that could provide refuge from pesticides are more likely to be potential sources of exposure in intensively managed agricultural landscapes^22^. As central place foragers, the reproduction of bees depends on the density and value of food resources within their foraging range^23–26^ and the proportion of the foraging range of a bee affected by pesticide use should correlate to their pesticide exposure^15,27,28^.

**Fig. 1:**
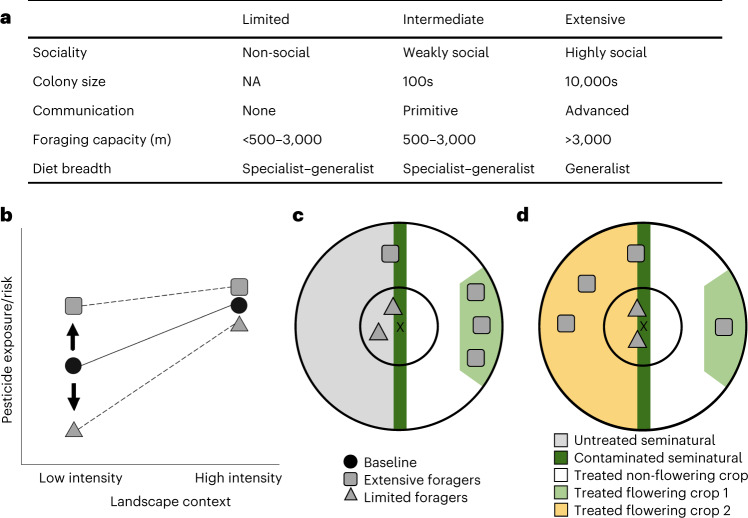
A trait-based, spatially explicit framework for the pesticide exposure and risk of bees. **a**–**d**, We describe three sets of foraging traits of bees (based on refs. ^23,30,82,83^)—‘extensive’, ‘intermediate’ and ‘limited’ (**a**), in relation to landscape context (**b**), as demonstrated in low-intensity (**c**) and high-intensity (**d**) landscapes, whereby extensive (grey square) and limited (grey triangle) foragers move between habitat types within their respective foraging ranges (concentric circles relative to X, the central nests). Our baseline assumption (**b**, black circles) is that pesticide exposure and risk will increase with agricultural intensification, proportional to the area of agricultural land within the foraging range of bees (**c** and **d**, concentric circles). We expect bees with the largest foraging range, ‘extensive’ foragers, to receive the highest pesticide exposure and risk independent of landscape context (**b**, line intercept; **c** and **d**, grey squares). However, as agriculture intensifies, the proportion of agricultural land within the foraging range of bees increases and the likelihood of foraging on contaminated food increases. Therefore, we expect ‘limited’ foragers to be disproportionately more at risk from pesticide exposure as agricultural land expands (**b**, line slope; **c** and **d**, grey triangles). NA, not applicable.

On the basis of the unique and correlated traits of bees, including sociality, communication, colony size, foraging capacity and diet breadth, we describe three sets of foraging traits: ‘extensive’, ‘intermediate’ and ‘limited’ (Fig. 1a). These traits will alter the pesticide exposure of bees in landscapes (Fig. 1b; line intercepts)^29^. For example, extensive foragers may be most exposed as they form large, highly eusocial colonies that communicate profitable, albeit potentially treated, mass-flowering crop resources which they can store for extended periods^30^. On the other hand, limited foragers do not accumulate extensive resources and are thus more reliant on seminatural habitats to provide continuous forage. Therefore, limited foragers may be less exposed if seminatural habitats are available and provide non-contaminated forage (compare ref. ^31^). However, limited foragers may become disproportionately more exposed in intensively managed agricultural landscapes, where there is an increased likelihood of contamination in the few seminatural habitats (Fig. 1b; line slope).

**Fig. 2:**
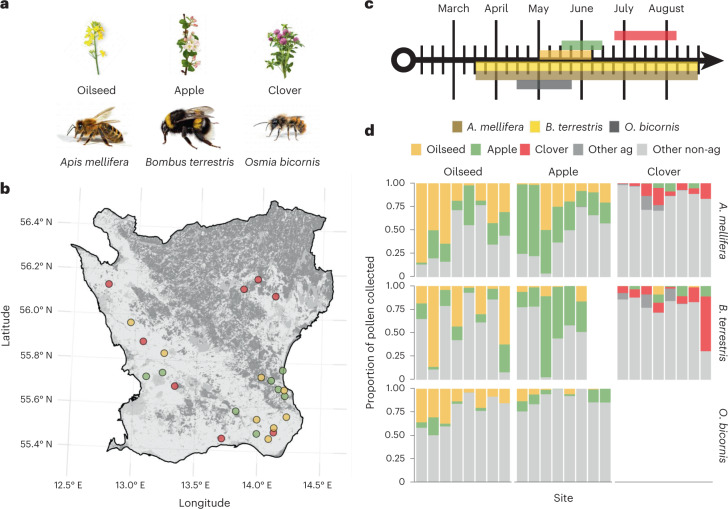
Study design for assessing bee pesticide exposure and risk in relation to different ecological traits and landscape contexts. **a**,**b**, We introduced sentinels of three bee species that vary in their sociality and foraging range to fields of three pollinator-dependent crops (**a**) across a gradient of land use in southernmost Sweden (**b**). Our focal bee species were *A. mellifera*, an extensive forager; *B. terrestris*, an intermediate forager; and *O. bicornis*, a limited forager. **c**, The activity periods and flowering phenology of bees and crops overlapped, except for red clover and *O. bicornis*. **d**, Non-agricultural (other non-ag) plant species/groups often dominated pollen use at each site (*x* axis) and bees tended to use more of the focal crop pollen than other agricultural (other ag) types. Pollen use and pesticide residue data are unavailable for red clover and *O. bicornis* due to non-overlapping phenologies (**c**). Due to colony failure, data are also absent for *B. terrestris* colonies at two apple sites. Images in **a** and map in **b** are free to use under creative commons licences (CC-BY and CC0).

To test whether foraging traits alter exposure and risk for bees in different landscape contexts, we assayed pesticide residues in pollen and nectar collected by *A. mellifera*, *B. terrestris* and *O. bicornis*, representing extensive, intermediate and limited foragers, respectively, across three sequentially blooming crops (Figs. 1 and 2). In doing so, we integrate multiple domains of pesticide exposure usually restricted to single studies: landscape context (for example, ref. ^32^), pollinator species (for example, ref. ^33^), crops (for example, ref. ^15^) and food sources (for example, ref. ^34^). We predicted that pesticide exposure and risk would increase with (1) the proportion of agricultural land and (2) the extent of foraging traits. Furthermore, we expected (3) limited foragers to experience greater pesticide exposure and risk than more extensive foragers with an increasing proportion of agricultural land. Additionally, we expected (4) that mass-flowering crops were the primary source of pesticide exposure, particularly for extensive foragers and that there may be crop-specific risks based on crop-specific pest management recommendations (Supplementary Table 1). Finally, we expected (5) pesticide exposure and risk to correlate between the pollen and nectar loads of bees, with potential application to postapproval pesticide monitoring. With expected drastic changes to pesticide regulation to meet current sustainability goals (for example, ref. ^35^) and calls for environmental risk assessment to become more accurate, reliable and holistic^36^, it is essential to understand why different cropping patterns and landscape contexts may differentially put key pollinator species at risk.

## Results

Across bee species (*A. mellifera*, *B. terrestris* and *O. bicornis*) and crops (oilseed rape, apple and clover) for both food sources (pollen and nectar), a total of 53 compounds were detected (of the 120 screened), including 24 fungicides, 19 herbicides, 5 insecticides, 2 acaricides, 2 metabolites of herbicides and 1 metabolite of a fungicide. We detected more compounds in pollen samples from oilseed rape sites (42, *n* = 40) than apple (36, *n* = 36) and clover sites (25, *n* = 32). The four compounds with the greatest compound-specific risk were insecticides (Table 1) but some herbicides and fungicides also ranked highly due to their high concentration or frequency (Supplementary Table 2). Herbicides and fungicides comprised 80% of total detections and 65% of total residues (in µg kg^−1^), yet unsurprisingly insecticides represented most of the pesticide risk, accounting for over 99% of the compound-specific risk (Supplementary Table 2).

**Table 1:** Compound-specific pesticide risk overall and for each bee species on the basis of the relevant detection rate and concentration (the latter is not shown for each species)

			Overall			*Apis mellifera*		*Bombus terrestris*		*Osmia bicornis*	
Pesticide (Type)	Pesticide group	LD_50_ mean	Concentration (90th)	Detections	Compound-specific risk	Detections	Compound-specific risk	Detections	Compound-specific risk	Detections	Compound-specific risk
Indoxacarb (I)	Oxadiazine	0.156	356	21 (34%)	775	9 (38%)	738	6 (27%)	1,070	6 (38%)	287
Imidacloprid (I)	Neonicotinoid	0.0420	4.50	3 (5%)	5.32	0 (0%)		1 (5%)	0.342	2 (12%)	14.0
Acetamiprid (I)	Neonicotinoid	11.3	65.5	47 (76%)	4.40	18 (75%)	2.99	14 (64%)	4.30	15 (94%)	5.02
Thiacloprid (I)	Neonicotinoid	28.1	66.0	56 (90%)	2.12	24 (100%)	2.82	20 (91%)	2.04	12 (75%)	3.09
Metamitron (H)	Triazinone	98.6^a^	36.9	44 (71%)	0.266	15 (62%)	0.113	14 (64%)	0.709	15 (94%)	0.261
Penconazole (F)	Triazole	7.10^a^	16.4	6 (10%)	0.231	3 (12%)	0.223	1 (5%)	0.167	2 (12%)	0.191
Tebuconazole (F)	Triazole	142^a^	117	16 (26%)	0.214	11 (46%)	0.0550	4 (18%)	4.57	1 (6%)	0.00200
Tau-fluvalinate (I)	Pyrethroid	12.3	14.0	4 (6%)	0.0680	4 (17%)	0.193	0 (0%)		0 (0%)	

^a^LD_50_ based on limit tests^75^.

Pesticide identity, type (I, insecticide; F, fungicide; H, herbicide; N, nematicide), group, toxicity (average acute and contact LD_50_ for *A. mellifera* adults, µg per bee^71^), concentration (90th percentile, µg kg^−1^), frequency of detection and compound-specific risk (Methods) of the five riskiest compounds for each species on the basis of their collected pollen and nectar.

**Fig. 3:**
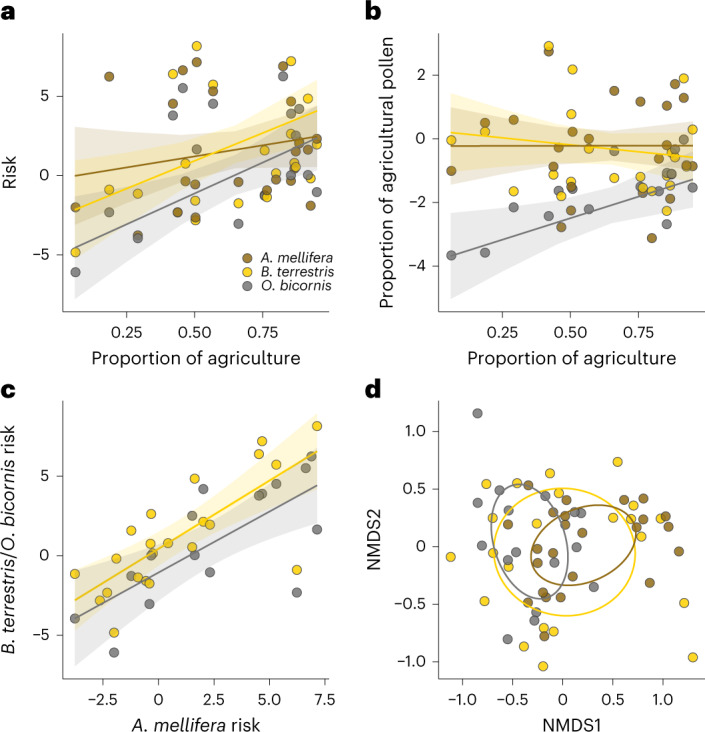
Pesticide risk and composition and agricultural pollen in relation to bee species and landscape context. **a**, Results showed that pollen-based pesticide risk increased with the amount of agricultural land in the landscape for *B. terrestris* and *O. bicornis*, while *A. mellifera* pollen-based risk was independent of agricultural land extent. **b**, The proportion of agricultural land also influenced pollen use, with only *O. bicornis* using more agricultural pollen with increasing agricultural land. **c**, Risk for *A. mellifera* correlated with that of *O. bicornis* (grey) and *B. terrestris* (yellow). **d**, The composition of pesticide compounds in pollen differed between *A. mellifera* and *O. bicornis*, while *B. terrestris* overlapped the two based on PERMANOVA of Bray–Curtis dissimilarities. Dispersion varied between bee species (*P* = 0.03); therefore, these community differences should be interpreted cautiously. Predictions and 95% confidence intervals (**a**,**b**,**c**) come from linear models with risk log transformed and the proportion agricultural pollen logit transformed. NMDS points (**d**) are based on standardised Bray–Curtis distances.

Pesticide risk (additive toxicity-weighted concentrations; Methods) was explained by the focal crop (*F*_2,20.48_ = 8.4, *P* < 0.01) and an interaction between bee species and the proportion of agricultural land in the landscape (Fig. 3a; *R*^2^m = 0.39, *F*_2,34.472_ = 4.4, *P* = 0.02) but not by an interaction between bee species and the focal crop (*F*_3,28.196_ = 0.1, *P* = 0.97) or the three-way interaction (*F*_3,28.10_ = 2.3, *P* = 0.10). The risk increased with the proportion of agricultural land for *O. bicornis* (trend estimate (confidence interval) 7.77 (2.53, 13.01)) and *B. terrestris* (7.00 (1.92, 12.08)), while that of *A. mellifera* (2.79 (−2.25, 7.83)) was independent of the proportion of agricultural land. The increase in risk was similar between *O. bicornis* and *B. terrestris* (Tukey-adjusted difference in slopes *P* = 0.91) but was stronger for *O. bicornis* than for *A. mellifera* (*P* = 0.03). The proportion of focal cropland (*F*_2,34.15_ = 1, *P* = 0.39) and mean-field size (*F*_2,34.35_ = 1.04, *P* = 0.36) in the 2 km radius landscape were not predictors of risk for any bee species.

The proportion of agricultural pollen collected by bees was also explained by focal crop (*F*_2,21.64_ = 9, *P* < 0.01) and an interaction between bee species and the proportion of agricultural land (Fig. 3b; *R*^2^m = 0.44, *F*_2,35.72_ = 4.4, *P* = 0.02), without an interaction between bee species and focal crop (*F*_3,28.70_ = 1.99, *P* = 0.14) or the three-way interaction (*F*_3,28.41_ = 1.35, *P* = 0.27). Agricultural pollen use by *O. bicornis* increased with the proportion of agriculture in the landscape (trend estimate 2.71 (0.55, 4.86)) but not for *A. mellifera* (0.01 (−1.93, 1.96) or *B. terrestris* (−0.88 (−2.87, 1.12)). On average, bees collected 30% oilseed rape-type pollen at oilseed rape sites, 29% apple-type pollen at apple sites and 12% clover-type pollen at red clover sites (Fig. 2d). The proportion of focal crop in the landscape did not influence the use of focal crop pollen by bees (*F*_2,35.01_ = 1.35, *P* = 0.27). Pesticide risk did not increase with the proportion of agricultural (*F*_2,35.28_ = 1.13, *P* = 0.33) or focal crop pollen (*F*_2,35.64_ = 1.40, *P* = 0.26).

**Fig. 4:**
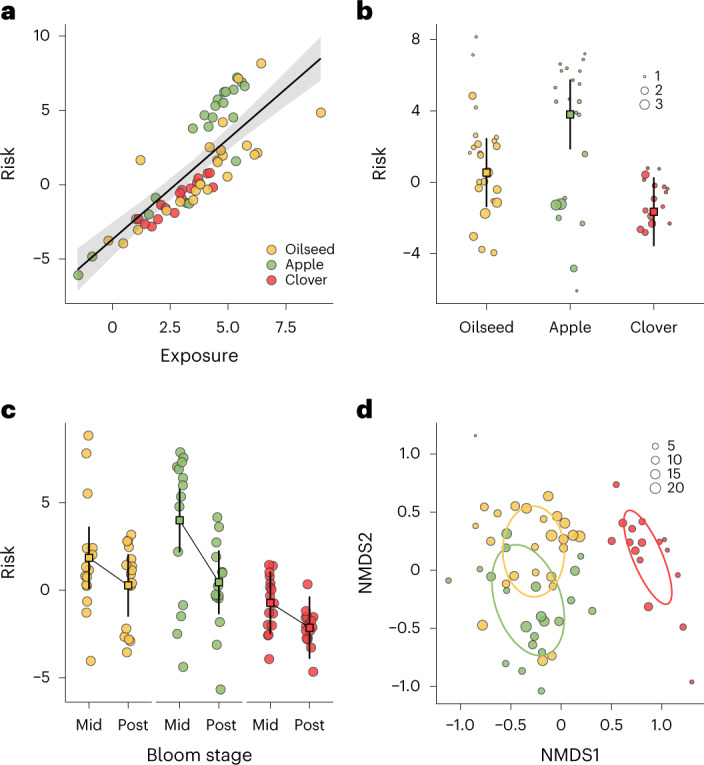
Pesticide risk and composition in relation to focal crop and bloom stage. **a**, Results show that pesticide risk and exposure were correlated (*R*^2^ = 0.74). **b**–**d**, Pollen-based risk (**b**), risk relative to the timing of focal crop bloom (**c**) and the composition of pesticide compounds (**d**) differed among focal crops on the basis of PERMANOVA of Bray–Curtis dissimilarities. We scaled points in **b** by their respective MCR (Methods), to depict pesticide mixture risk relative to its constituent single most risky compound. A value close to one indicates that a single compound dominates the mixture risk. MCR values did not differ between crops (Supplementary Fig. 1). Outlined squares (**b** and **c**) depict means and 95% confidence intervals (**b**, oilseed rape *n* = 24, apple *n* = 22 and clover *n* = 16; **d**, oilseed rape *n* = 32, apple *n* = 28 and clover *n* = 32). We scaled points in **d** by the number of pesticides detected in a pollen sample. Predictions and 95% confidence intervals (**a**,**b**,**c**) come from linear models with risk log transformed. NMDS points (**d**) are based on standardised Bray–Curtis distances.

We found that bee species experienced similar site-level risk—*A. mellifera* related to *B. terrestris* (Fig. 3c; *R*^2^ = 0.6, *T* = 4.19, d.f. = 18, *P* < 0.01) and *O. bicornis* (Fig. 3c; *R*^2^ = 0.53, *T* = 3.57, d.f. = 13, *P* < 0.01) and *O. bicornis* related to *B. terrestris* (*R*^2^ = 0.65, *T* = 4.48, d.f. = 11, *P* < 0.01). Pesticide risk and exposure were correlated (Fig. 4a; *R*^2^m = 0.74, *F*_1,55.92_ = 111.31, *P* < 0.01) and we provide parallel exposure results (additive concentrations) in the Supplementary Results.

Pollen collected at apple sites had higher risk compared to clover sites (Fig. 4b; *T* = 4.09, d.f. = 21.2, *P* < 0.01) but was similar between oilseed rape and apple sites (*T* = **−**2.39, d.f. = 19.5, *P* = 0.07) and oilseed rape and clover sites (*T* = 1.69, d.f. = 20.8, *P* = 0.23) (Fig. 4b). Risk (Fig. 4c) and exposure (Supplementary Fig. 2) were higher during crop bloom than after crop bloom.

Compound composition in pollen differed between the focal crops (PERMANOVA *F*_2,61_ = 11.34, *P* < 0.01) and between bee species (PERMANOVA *F*_2,61_ = 2.12, *P* = 0.01), without an interaction between bee species and focal crop (*P* > 0.05). Between bee species, the compound composition only differed between *O. bicornis* and *A. mellifera* (Fig. 3d and Supplementary Table 3; *F*_1,38_ = 3.85, *P* < 0.01). Between focal crops, all pairwise comparisons indicated different compound compositions (Fig. 4d and Supplementary Table 3, all *P* < 0.01).

**Fig. 5:**
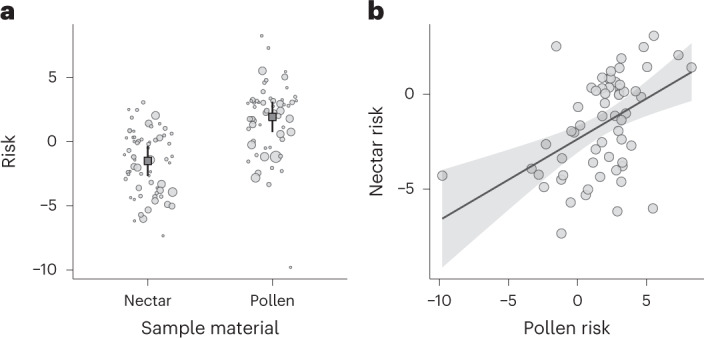
Pesticide risk in bee-collected pollen and nectar. **a**, Results show that pesticide risk was greater from pollen than nectar, but the risk correlated between sample materials. As in Fig. 4b, points in **a** are scaled by their respective MCR, where a smaller point indicates that a single compound dominates the pesticide mixture risk. Outlined squares (**a**) depict mean log transformed risk (nectar *n* = 70 and pollen *n* = 61). Predictions and 95% confidence intervals (**a**,**b**) come from linear mixed-effects models with risk log transformed. **b**, We re-analysed data with the left-hand outlier removed and the results were qualitatively unchanged and the model fit improved.

Risk, not accounting for assumptions of residue intake, for example, via consumption, was higher in pollen than in nectar (Fig. 5a; *T* = −10.66, d.f. = 93.9, *P* < 0.01) and the pesticide composition differed between these sample materials (Supplementary Fig. 3, PERMANOVA *F*_1, 49_ = 2.42, *P* = 0.04). We found that the pollen-based risk related to the nectar-based risk (Fig. 5b; *R*^2^m = 0.10, *T* = 2.15, d.f. = 53.99, *P* = 0.04).

## Discussion

The pesticide exposure of bees arises from their activity intersecting pesticide use^12^. Thus, pesticide exposure and its correlated risk (additive toxicity-weighted concentrations) to bees are likely to be affected by their life-history traits^37^, particularly foraging habits^23,26,38^ and land-use and pesticide-use patterns, especially in bee-attractive crops^39,40^. Using an ecological approach to pesticide risk, we found that extensive foragers (*A. mellifera*) experienced the greatest risk irrespective of the proportion of agricultural land in the landscape. Although risk correlated among bee species, both limited foragers (*O. bicornis*) and intermediate foragers (*B. terrestris*) experienced less risk than extensive foragers (*A. mellifera*) in landscapes with less agricultural land. In addition, risk correlated between sample materials and was greatest in pollen. Consequently, *A. mellifera*-collected pollen can cautiously predict pesticide risk for bees, not accounting for residue intake, compared to nectar and pollen collected by other bee species, independent of landscape context. Thus, the *A. mellifera*-collected pollen-based pesticide risk indicator may be a promising metric for postapproval pesticide monitoring in terrestrial systems, generally proposed by ref. ^41^ and with parallels in aquatic systems^42^.

Agricultural landscapes expose *A. mellifera* to multiple pesticides^15,27,32,43–45^. However, we know less about the resulting pesticide risk, especially between bee species and in different landscape contexts (but see refs. ^22,26,46,47^). We found that increasing the proportion of agricultural land increased the risk for *B. terrestris* and *O. bicornis* but not for *A. mellifera*. We suggest that these landscape-dependent differences in risk result from species-specific activity patterns^23,38^. Different crop pollen use between the three species somewhat supports this: uniform collection by *A. mellifera* and *B. terrestris* compared to increasing collection by *O. bicornis* with an increasing proportion of agricultural land, consistent with findings in apple for *A. mellifera*^32^ and *O. cornifrons*^28^. Consequently, mass-flowering crops appear to be a predominant food source for *A. mellifera* across agricultural landscapes^32,38,40,45^. In contrast, despite access to mass-flowering crops, *O. bicornis* favours non-crop, predominantly woody, pollen resources when available^48,49^. These different preferences for crop pollen are evidenced by others finding that the collection of focal crop pollen positively correlates to the proportion of that crop in the landscape for *A. mellifera* (apple^32^) and *B. impatiens* (blueberry^27^) but not *O. bicornis* (oilseed rape^31,48^). Therefore, sets of foraging traits (for example, large colony size and advanced communication) and foraging preferences probably drive the prevalence of *A. mellifera* in mass-flowering crops. In intensively managed agricultural landscapes with scarce seminatural habitats and high pesticide use, *O. bicornis* is increasingly likely to forage in less-preferred mass-flowering crops and seminatural habitats adjacent to arable land^31^ and thus increase their pesticide exposure and risk. Consequently, populations of *O. bicornis* and similar, limited foragers may be disproportionately affected by agricultural intensification as their traits compound the combined effects of habitat loss and increased pesticide exposure^26^. Our use of *O. bicornis* as a sentinel allowed us to estimate exposure and risk of a limited forager in landscapes where they may not naturally occur, which, combined with the relatively generalised diet of *Osmia* spp.^26,48,50^, means that our estimates for limited foragers are probably precautionary among solitary bee species.

The focal crop (oilseed rape, apple or clover) was an important driver of pollen-derived exposure and risk for all bee species, independent of the proportion of agricultural land. For example, all bee species experienced the highest exposure and risk at apple sites, followed by oilseed rape and clover sites. These results mirror the approved number of active ingredients in plant protection products recommended for use in the three focal crops, with most in apple and least in clover. Apple and other fruit crops generally have higher pesticide use^51^ and resulting bee exposure than annual arable crops or permanent grasslands^15^. We also found that the composition of pesticides in pollen differed between the three crops, identifying pest management strategies for specific crops and even specific compounds as determinants of landscape-level exposure and risk. Pollen pesticide risk was greater during crop bloom than after bloom across all three investigated crops. However, it did not correlate with either agricultural or focal crop pollen collection, possibly pointing toward the treated crop and associated flowering plants affected by drift as sources of pesticide exposure^20,22,26,27^. Focusing on spatiotemporally matched pollen and nectar samples from *A. mellifera* and *B. terrestris*, we found that exposure and risk were higher in pollen than in nectar, although this does not account for the uptake of residues by bees for example via consumption which is unequal between pollen and nectar^33^. Nonetheless, we found that risk but not exposure positively correlated between pollen and nectar; thus, pollen may be a precautionary material for estimating the pesticide risk of bees and, more generally, pesticide contamination of terrestrial environments^34,52^.

Pollen pesticide mixture composition differed the most between *A. mellifera* and *O. bicornis*, while *B. terrestris* overlapped the two. The three species shared two of the riskiest compounds, indoxacarb and acetamiprid, while the following most risky compounds were unique to each species: thiacloprid for *A. mellifera*, tebuconazole for *B. terrestris* and imidacloprid for *O. bicornis*. Nevertheless, risk positively correlated among the three species, suggesting that risk estimates for one species can, to some degree, inform the risk to other bee species. The generally low maximum cumulative ratio (MCR) values indicate that the pesticide mixture risk, independent of bee species and focal crop, was driven by one or a few high-risk compounds (similar to ref. ^53^). High-risk compounds were mainly neonicotinoid insecticides (acetamiprid, imidacloprid and thiacloprid), previously identified as high-risk to bees^33,54^ but the riskiest compound was indoxacarb, an oxadiazine insecticide. Reduced exposure to these high-risk compounds would substantially decrease the risk for the three bee species. In the EU, pesticide restrictions (imidacloprid 2018, thiacloprid 2021 and indoxacarb 2022) are regulatory moves in this direction^55–57^, even if residues persist (like imidacloprid in our study^58^) or new compounds with similar risk profiles enter the market in the future^59,60^.

Pesticide risk assessment primarily focuses on *A. mellifera*, partly because of its economic value, ease of management and a greater understanding of the species’ biology^61–63^. However, risk assessment is becoming more holistic^36^, with a greater emphasis on non-*Apis* species^64^ in recognition of wild bee diversity and their contribution to pollination services^65^. However, this change requires a better understanding of how pesticide risk varies among bee species and landscape contexts. We found that the pesticide risk estimated from *A. mellifera*-collected pollen was generally higher than or similar to *B. terrestris* and *O. bicornis*, particularly in landscapes with less agricultural land. Thus, whilst bee traits regulate pesticide exposure and risk, there is potential to extrapolate risk among bee species and exposure sources, with higher and thus precautionary risk estimates based on *A. mellifera*-collected pollen. However, pesticide exposure and our ecological indicator of pesticide risk do not account for species-specific processes past the pesticide use–bee activity intersection, such as consumption within the nest or indirect effects that could affect the fitness of the bees—important considerations when moving from exposure to effect in environmental risk assessment^63^.

Using our trait-based approach, we conclude that landscape context modifies pesticide risk but only for limited and intermediate foragers (here, *O. bicornis* and *B. terrestris*, respectively). These findings highlight the potential for seminatural habitats to buffer pesticide-related risks for wild bees^26,46,66^. We also conclude that *A. mellifera*-collected pollen can predict environmental pesticide risk for other species and is precautionary, particularly in less agriculturally dominated landscapes. We, therefore, suggest that an *A.* mellifera-collected pollen-based pesticide risk indicator is a promising metric for postapproval pesticide monitoring in terrestrial systems (compare ref. ^41^). However, questions remain as to how this exposure affects individuals and, ultimately, populations of bees—tasks for a more holistic and realistic environmental risk assessment that aims to capture exposure to pesticide mixtures and risks within the diverse bee community^67^.

## Methods

### Field site system and sentinel bees

We centred 24 sites on three bee-attractive flowering crops: oilseed rape (8 sites), apple (8 sites) and red clover grown for seed production (8 sites) in southern Sweden (Fig. 2). These crops bloom sequentially: oilseed rape during April–May, apple during May–June and red clover during June–August (Fig. 2c) and are affected by different pests and therefore have different pest management strategies. The national pest management recommendations for 2019 included 26 active ingredients in oilseed rape, 32 in apple and 14 in clover seed and included acaricide (2 active ingredients), fungicide (20), herbicide (20) and insecticide (13) products (Supplementary Table 1). We selected sites on the basis of their surrounding proportion of agricultural land (2 km radius) to ensure an even gradient (for each crop type) of agricultural land and, therefore, anticipated pesticide use^15,16,68^. The average (± s.d.) proportion of agricultural land was 74 ± 24% (range 29–95%) for oilseed rape, 52 ± 29% (6–85%) for apple and 66 ± 20% (44–93%) for clover. All sites were >6 km apart, except for two clover sites, 2 km apart. Southern Sweden is characterised by annual crop production and nationally high pesticide use^69^. Farmers managed crops conventionally, except for one field of each focal crop, which was managed organically.

In 2019, we placed sentinel bees at focal crop fields at the onset of flowering and allowed them to forage freely without supplemental food. At each field, we placed: (1) two or three nationally produced, standardised and conventionally managed *A. mellifera* colonies, (2) six commercial *B. terrestris* colonies (Biobest Biological Systems) in two large ventilated wooden boxes and (3) three solitary bee trap nest units (at the oilseed rape and apple sites) each seeded with 50 male and 50 female *O. bicornis* cocoons (Wildbiene & Partner) (Supplementary Methods). We did not place *O. bicornis* in clover fields as their phenologies do not overlap (Fig. 2c).

### Quantification of pesticide residues in pollen and nectar

We sampled pollen from (1) *A. mellifera* using pollen traps attached to two hives for 24 h, (2) *B. terrestris* by capturing foragers (~20 across all six colonies) and killing them on dry ice as they returned to their colonies and (3) multiple *O. bicornis* brood cell pollen provisions collected by females over the second half of the bloom period. We sampled pollen from *A. mellifera* and *B. terrestris* at two sampling intervals, coinciding with (1) the peak of crop bloom and (2) after crop bloom and for *O. bicornis* only at the end of crop bloom (evenly from all the available pollen). In total, we collected 48 samples (595 g) of *A. mellifera*-collected pollen, 44 samples (11 g) of *B. terrestris-*collected pollen and 16 samples (70 g) of *O. bicornis*-collected pollen. During and after bloom, samples were pooled for both *A. mellifera* and *B. terrestris*, resulting in 24 samples of *A. mellifera*-collected pollen, 22 samples of *B. terrestris*-collected pollen (all colonies died at two sites) and 16 samples of *O. bicornis-*collected pollen. We did not pool *O. bicornis* pollen over the bloom period since this species already combined pollen provisions on our behalf.

To compare residues between nectar and pollen, we sampled additional returning foragers of *A. mellifera* (*n* ≈ 100 individuals per sample) and *B. terrestris* (*n* ≈ 20 individuals per sample) 1–2, 4–6 and 12–16 days after a known pesticide application at four oilseed rape, two apple and seven clover sites (Supplementary Table 4). Corbicular pollen and nectar stomach content were collected from these foragers to produce paired pollen and nectar samples for each site and collection time point (*n* = 54).

We froze pollen and bee samples, before nectar extraction, at −20 °C before screening for 120 pesticide compounds included in the Swedish national monitoring scheme (Supplementary Table 5), following established protocols at the Laboratory for Organic Environmental Chemistry (SLU) (Supplementary Methods).

### Pollen identification

Part of each pollen sample was analysed to determine the pollen use of the three bee species at each site. First, we pooled pollen samples per site, bee species and bloom period in a 5 ml tube and agitated them in 5 ml of 70% ethanol before pipetting 2 μl of the pollen suspension onto a microscope slide stained and set using fuchsin gel under a coverslip. Next, we identified (using a pollen reference library at the Department of Biology (Lund) and ref. ^70^) and counted >400 pollen grains per slide (7–20 rows, 163 μm wide across the slide) using ×400 magnification. On the basis of this, we quantified the proportional use of all agricultural-type pollen and focal crop pollen by bees and categorised the latter into a Brassicacae group (including oilseed rape; *Brassica napus*)*, Malus* group (including apple; *Malus domestica*) and *Trifolium pratense* group (including red clover; *T. pratense*) (Supplementary Table 6).

### Landscape classification

We analysed the landscape surrounding our sites at multiple spatial scales (1,000, 1,500 and 2,000 m, corresponding to the average foraging capacities of bees (Fig. 1a)) on the basis of the IACS Spatial Data Layer provided by the Swedish Board of Agriculture. We classified land cover categories into two groups: agricultural land (all types of agricultural use, such as annual crops, orchards, leys and seminatural grasslands) and non-agricultural land (including forest, urban areas and water bodies). This distinction is because our focus was on the pesticide exposure and risk to bees from agricultural pesticide use and the pesticide exposure of bees is higher in rural compared to urban areas^22^. We also calculated the proportion of the focal crop in the radii and the average field size. We confirmed that the proportion of agricultural land was consistent (Supplementary Fig. 8) and correlated (Supplementary Fig. 9) across the three spatial scales for each crop type and consequently used the landscape information at the largest scale (2,000 m) in all subsequent analyses.

### Risk calculations

We use toxicity-weighted concentrations (TWC) as a basis for indicating pesticide risk for bees^26^, where the TWC of each compound (TWC_*i*_) is the ratio between the concentration (*c*_*i*_) of a detected compound in bee-collected pollen or nectar and its respective acute toxicity endpoint (LD_50*i*_—the dose required to cause 50% mortality in the test population)^71^. Then, following a concentration addition approach—the recommended default for mixture environmental risk assessment^72^ (even though some compound classes may synergize^73^), we summed TWCs, to calculate the additive toxicity-weighted concentration of all compounds within a sample per site and bee species (TWC_mix_):

$$\mathrm{TWC}_\mathrm{mix} = \mathop {\sum }\limits_{i = 1}^{n} \frac{{c_{i}}}{{\mathrm{LD}_{50}}{i}}$$

Henceforth, we refer to this metric, an indicator of pesticide-related risk, as ‘risk’.

We averaged the acute oral and contact LD_50_ (ref. ^71^) of each compound to provide an overall indicator of toxicity, reflective of how bees encounter pesticides in the landscape and their multiple exposure routes^37^. We used LD_50_ for adult *A. mellifera* because there are incomplete toxicity data for other bee species and life stages and, where there are data, LD_50_ for other bee species correlate with the corresponding *A. mellifera* LD_50_ (refs. ^53,74^). Furthermore, in using the same LD_50_ across bee species, we disentangle the ecology of bees from toxicology to explore relative differences in the activity patterns of bees in intersection with pesticide use. Finally, we used the tested dose for LD_50_ based on limit tests^71^ (used when a compound is expected to be low in toxicity or there are issues with solubility^75^), which can overestimate the toxicity of a compound. Three of these compounds ranked highly for compound-specific risk due to their high concentrations and frequency of detection rather than toxicity (Table 1).

We also calculated the factor by which the mixture risk (TWC_mix_) was greater than its composite most risky compound (max(TWC_*i*_)) using an MCR^76^. Thus, an MCR close to one indicates that a single compound dominates risk. The MCR did not vary among bee species or focal crops (Supplementary Fig. 1).

Finally, we also calculated compound-specific risk (Table 1 and Supplementary Table 2) to identify high-risk compounds by multiplying TWC_*i*_ by its bee-specific detection frequency^33^.

### Data analyses

We conducted four primary analyses to understand agricultural pesticide risk to bee species, followed by supporting multivariate analyses of the compound compositions. We performed analyses and data visualization using R v.4.1.1., constructed linear mixed-effects models (LMMs) with the lme4 package^77^ and analysed compound composition with the vegan package^78^. For the primary analyses, risk data were log transformed and the proportion of crop pollen was logit transformed to meet assumptions of normality and homogeneity of variance. Upon detecting significant main effects, we examined the significance and difference of individual factor levels via pairwise comparisons of estimated marginal means using Tukey’s method with the emmeans package^79^. Finally, we evaluated models for overdispersion and checked residuals for normality and homoscedasticity using diagnostic functions in the performance package^80^. We report marginal *R*^2^ values calculated following the methods of ref. ^81^.

#### Risk and pollen use with landscape context, focal crop and bee species

We used LMMs to explore (1) risk from pollen and (2) use of agricultural pollen, with focal crop and bee species interacting with the proportion of agricultural land as fixed effects and site as a random intercept. We included an interaction between bee species and crop for both analyses but this was non-significant and thus removed. Additionally, we used a similar model, including the focal crop, bee species interaction and site as random intercept, to relate focal crop pollen to the proportion of that focal crop in the landscape.

#### Risk with sampling round and focal crop

We tested whether risk varied between the different sampling rounds using an LMM with sample round, focal crop and bee species included as fixed effects and site as a random intercept. Finally, we tested if risk related to the proportion of focal crop pollen, bee species and focal crop, with focal crop pollen interacting with bee species, as fixed effects and site as a random intercept.

#### Risk among bee species

We examined risk relationships among the site-specific pollen collection of bee species using three linear models, one for each species. We included the remaining bee species and focal crop as fixed effects; however, the focal crop was non-significant in all models (*P* > 0.05).

#### Risk between sample materials

We used data from the paired pollen–nectar collections to test for a difference in risk between sample materials (pollen versus nectar), using LMMs with sample material, focal crop and bee species as fixed effects, and sampling round nested in the site as a random intercept. In addition, we examined risk relationships among sample material collections, using an LMM with nectar risk specified as the response variable and pollen risk, focal crop and bee species as fixed effects, and sampling round nested in the site as a random intercept.

#### Differences in compound composition

We used PERMANOVA to compare the composition of compounds between focal crops and bee species using a Bray–Curtis dissimilarity index based on a Hellinger standardised community matrix of risk values using the adonis2() function in vegan. We used non-metric multidimensional scaling (NMDS) to visualise different clusters of compounds. We tested for differences in dispersion between focal crops or bee species using the betadisper() function in vegan. We detected no differences in the dispersion of compounds between crops. However, we found different dispersion of compounds between bee species (*P* = 0.03); therefore, we should interpret these community differences cautiously.

### Reporting summary

Further information on research design is available in the Nature Portfolio Reporting Summary linked to this article.

## Supplementary information

Supplementary Information Supplementary Methods, Results, Figs. 1–9 and Tables 1, 3, 4 and 6.

Reporting Summary

Peer Review File

Supplementary Tables Supplementary Table 2 lists all detected compounds, their frequency, mean LD50 and associated risk. Supplementary Table 5 lists all screened compounds and estimated detection limits (LODs).

## Data Availability

Data available via Figshare 10.6084/m9.figshare.20390751.
