# Supplementary material for: Ecological traits interact with landscape context to determine bees’ pesticide risk
Source: Nat Ecol Evol. 2023 Feb 27;7(4):547–56. doi: 10.1038/s41559-023-01990-5 (PMC10089916; doi:10.1038/s41559-023-01990-5)
Supplement: Supplementary file 1 — Supplementary Methods, Results, Figs. 1–9 and Tables 1, 3, 4 and 6. [file 41559_2023_1990_MOESM1_ESM.pdf]

nature ecology & evolution

Article https://doi.org/10.1038/s41559-023-01990-5

Ecological traits interact with landscape context to determine bees' pesticide risk

In the format provided by the authors and unedited

Supplementary Information

ECOLOGICAL TRAITS INTERACT WITH LANDSCAPE CONTEXT TO DETERMINE BEES' PESTICIDE RISK

Jessica Knapp (knappj@tcd.ie), Charlie C. Nicholson, Ove Jonsson, Joachim R. de Miranda, and Maj Rundlöf (maj.rundlof@biol.lu.se)

Table of Contents

Ecological traits interact with landscape context to determine bees' pesticide risk 1
Supplementary methods 2
  Field site system and sentinel bees 2
  Quantification of pesticide residues in pollen and nectar 3
Supplementary results 4
  Exposure and pollen use with landscape and bee species 4
  Exposure among bee species 4
  Exposure between sample materials 4
Supplementary figures 5
  Figure S2. 6
  Figure S3. 7
  Figure S4. 8
  Figure S5. 9
  Figure S6. 10
  Figure S7. 11
  Figure S8. 12
  Figure S9. 13
Supplementary tables 14
  Table S1. 14
  Table S2. 16
  Table S3 17
  Table S4. 18
  Table S5. 19
  Table S6. 20

SUPPLEMENTARY METHODS

Field site system and sentinel bees

The *Apis mellifera* colonies were prepared at the end of April 2019 in local Swedish colony size (Lågnormal; inside dimensions, 382 x 382 x 230 mm; about ¾ the size of a full-frame Langstroth hive) with two frames of brood, two frames of nectar and pollen stores, four frames of drawn comb and two frames of foundation; about 0,5 kg bees and a laying, open-mated 1-2-year-old queen of mixed genetic stock (primarily *A.m. carnica* with traces of *A.m. ligustica* and *A.m. mellifera*). The colonies were treated for varroa with two strips of Apistan (tau-fluvalinate) between 1 September-13 October 2018 and a single treatment of 3.2% oxalic acid in sugar syrup in November 2018. Varroa treatment was not applied during the 2019 experiments, although varroa development was monitored. The colonies were free from American foulbrood (AFB), European foulbrood (EFB) and tracheal mites (*Acarapis woodi*), the three primary reportable diseases in Sweden. We supplied the colonies with extra space as required and managed to prevent swarming. None of the colonies swarmed during the experiments, although one colony did lose its queen, which we did not replace.

Standard colonies of *Bombus terrestris* were sourced from Biobest Biological systems (Belgium). Each colony contained a queen and about 80 worker bees plus brood. We removed the sugar water provision to make the bees forage for nectar and pollen, i.e. to resemble foraging in wild bumblebees.

Cocoons of *Osmia bicornis* were sourced from Wildbiene & Partner (Switzerland) and stored hibernating at 4°C before a diapause break at 10°C. The cocoons were then placed in an emergence tube within the nesting unit for release. The nesting units were designed by Red BeeHive (UK) and consisted of three plastic trap nests filled with a central emergence tube surrounded by cardboard nesting tubes, mounted on a wooden pole at 1-1.5 m high off the ground.

Quantification of pesticide residues in pollen and nectar

Pollen and bee samples (for subsequent collection and analysis of nectar) were sent on dry ice to the Laboratory for Organic Environmental Chemistry at SLU and frozen at -20 °C pending analysis. Pollen samples were homogenised and 0.20 g extracted with acetonitrile, first in 7 mL Precellys mixing tubes containing ceramic beads (Bertin instruments), then by ultra-sonication using a Vibracell VCX 130 instrument with a 6 mm sonication probe from Sonics. The combined extract was split in two, one fraction for determination with liquid chromatography-tandem mass spectrometry LC-MS/MS (Agilent 1260 Infinity pump system connected to an Agilent 6460 triple quad mass spectrometer), the other fraction, further cleaned with dispersive solid phase extraction ($MgSO_4$, C18 and primary/secondary amine, Part No. KS0-8921, Phenomenex) for gas chromatography-mass spectrometry with negative chemical ionisation GC-(NCI)MS (Agilent 7890A GC connected to a 5975C mass spectrometer). Nectar was collected from the honey stomachs of 20 dead bees for each sample, and a 20 µL aliquot was prepared for LC-MS/MS analysis using protein precipitation with acetonitrile (1:4, v:v). Internal standard compounds for LC and GC target compounds were added to all samples before extraction (pollen) or in connection with protein precipitation (nectar). Method performance was controlled using fortified pollen and nectar samples, from which relative recoveries (i.e. detector signals of target compounds relative to internal standard signals) were determined and used for concentration calculations. All analytical batches included blank matrix samples, method blanks and calibration samples at 6-8 concentration levels.

SUPPLEMENTARY RESULTS

Exposure and pollen use with landscape and bee species

As with pesticide risk, exposure was explained by focal crop (Fig. S4; $F_{2, 21.13} = 7.4$, $P < 0.01$) and an interaction between bee species and the proportion of agricultural land in the landscape (Fig. S5; R^2m = 0.54, $F_{2,35.15} = 3.3$, $P = 0.05$), but not by an interaction between bee species and focal crop ($F_{3,28.47} = 1.4$, $P = 0.24$) or the three-way interaction ($F_{3,28.27} = 2.3$, $P = 0.10$). Exposure increased with the proportion of agricultural land for *O. bicornis* (trend estimate [CI]: 5.41 [2.89, 7.92]), *B. terrestris* (5.96 [3.60, 8.33]), and *A. mellifera* (3.06 [0.74, 5.38]). The increase in exposure was similar between all species (Tukey-adjusted difference in slopes $P > 0.05$)

Exposure in pollen collected at apple sites was greater than at clover sites ($T = 3.8$, df = 23.1, $P < 0.01$) (Fig. S4). Pesticide exposure was comparable between oilseed rape and apple (Fig. S4; $T = -1.48$, df = 18.7, $P = 0.32$) and oilseed rape and clover (Fig. S4; $T = 2.41$, df = 22.1, $P = 0.06$).

Exposure among bee species

We found that the pesticide exposure experienced by *A. mellifera* was related to *B. terrestris* exposure (Fig. S6; $R^2 = 0.50$, $t = 3.067$, df = 18, $P < 0.01$) and *O. bicornis* exposure (Fig. S6; $R^2 = 0.45$, $T = 3.22$, df = 13, $P < 0.01$). *O. bicornis* and *B. terrestris* exposure were also correlated ($R^2 = 0.62$, $T = 4.23$, df = 11, $P < 0.01$).

Exposure between sample materials

We found higher exposure in pollen than in nectar (Fig. S7*a*; $T = -10.20$, df = 94.2, $P < 0.01$). We found that the pollen-based exposure was not predictive of nectar-based exposure (Fig. S7b; R^2m = 0.09, $T = 1.67$, df = 53.59, $P = 0.10$).

SUPPLEMENTARY FIGURES

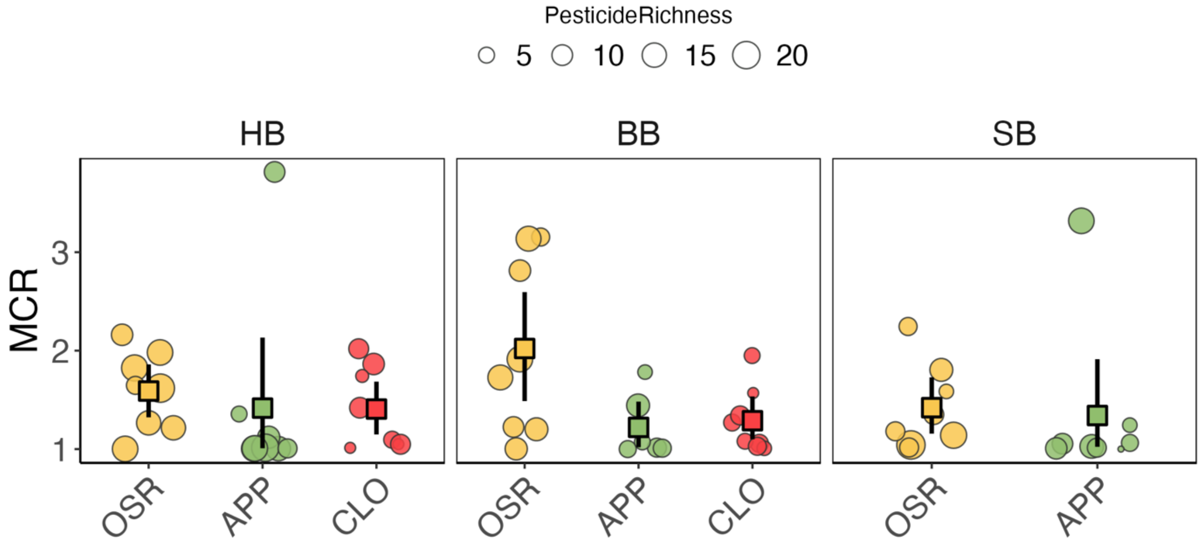

Figure S1. Maximum cumulative ratio (MCR) between focal crops (APP: apple; CLO: clover; OSR: oilseed) and bee species (HB: Apis mellifera; BB: Bombus terrestris; SB: Osmia bicornis). Values of MCR are the ratio of the toxicity-weighted exposure of the mixture to the highest toxicity-weighted exposure of a single compound (Price & Han 2011). Thus, MCR values are the factor by which the mixture is riskier than its constituent single most risky compound; thus, a value close to one indicates that a single compound dominates the mixture risk. There are no differences between MCR values based on linear mixed effects models with an interaction between crop and bee species and the site as a random intercept ($P < 0.05$). Jittered points scale the number of pesticides occurring in the pollen mixture. Outlined points depict means and 95% confidence intervals.

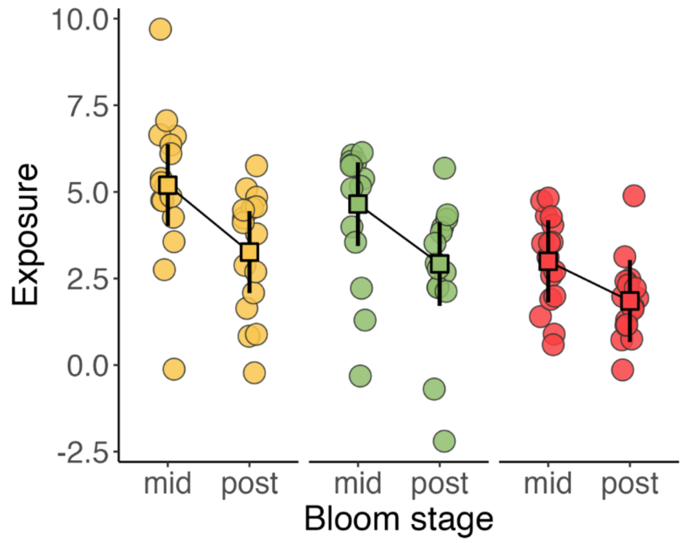

Figure S2. Pesticide exposure was greatest during crop bloom (all pairwise differences within the focal crop, oilseed (yellow), apple (green), and clover (red), significant at $P < 0.05$). Predictions and 95% confidence intervals are from linear mixed effects models with exposure log transformed.

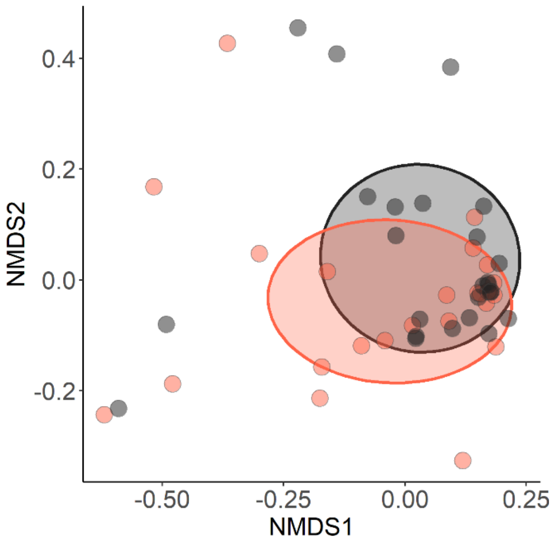

Figure S3. Pesticide composition differed between sample materials: pollen (red) and nectar (grey). We base points in the NMDS plot on standardised Bray-Curtis distances.

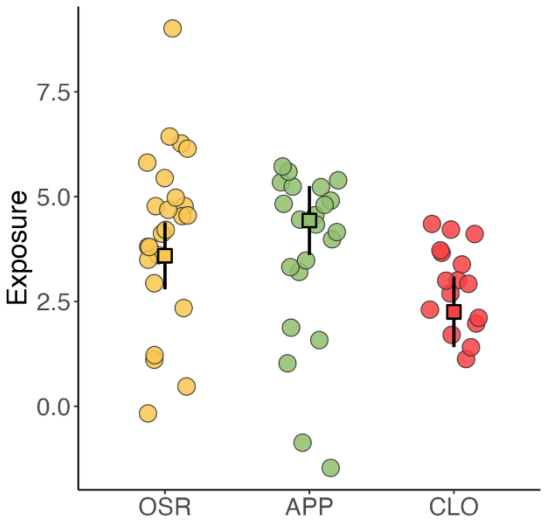

Figure S4. Pesticide exposure in pollen differed between cropping systems (APP: apple; CLO: clover; OSR: oilseed rape). Error bars depict 95% confidence intervals.

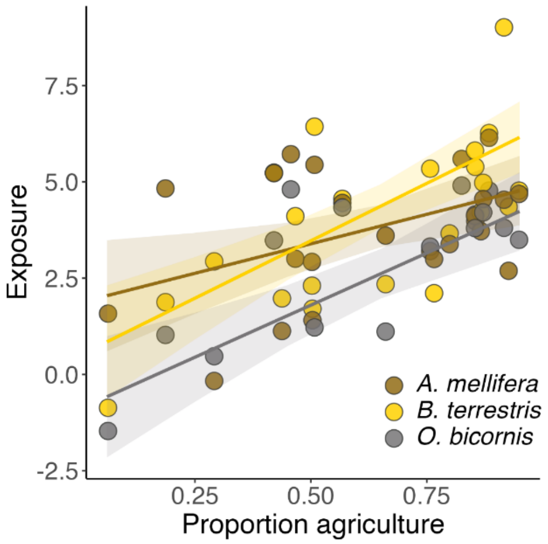

Figure S5. Exposure to pesticide residues in pollen increased with the amount of agricultural land surrounding focal fields at a similar rate for the three bee species. Predictions and 95% confidence intervals are from a mixed effects model.

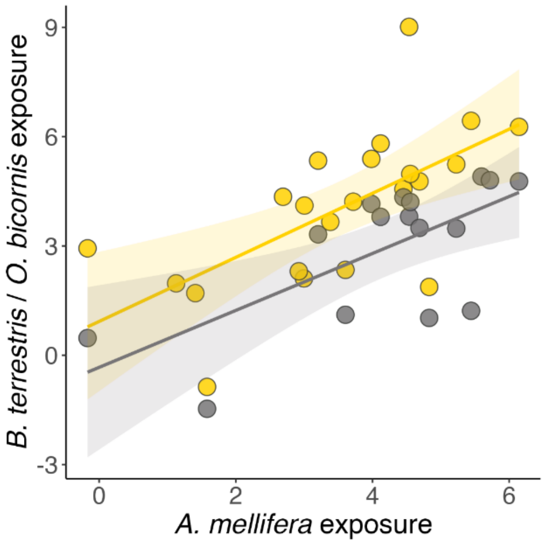

Figure S6. Exposure from pesticide residues in *A. mellifera* pollen correlated with exposure in *O. bicornis* (black) and *B. terrestris* (yellow) pollen samples predictions, and 95% confidence intervals come from linear models with exposure log transformed.

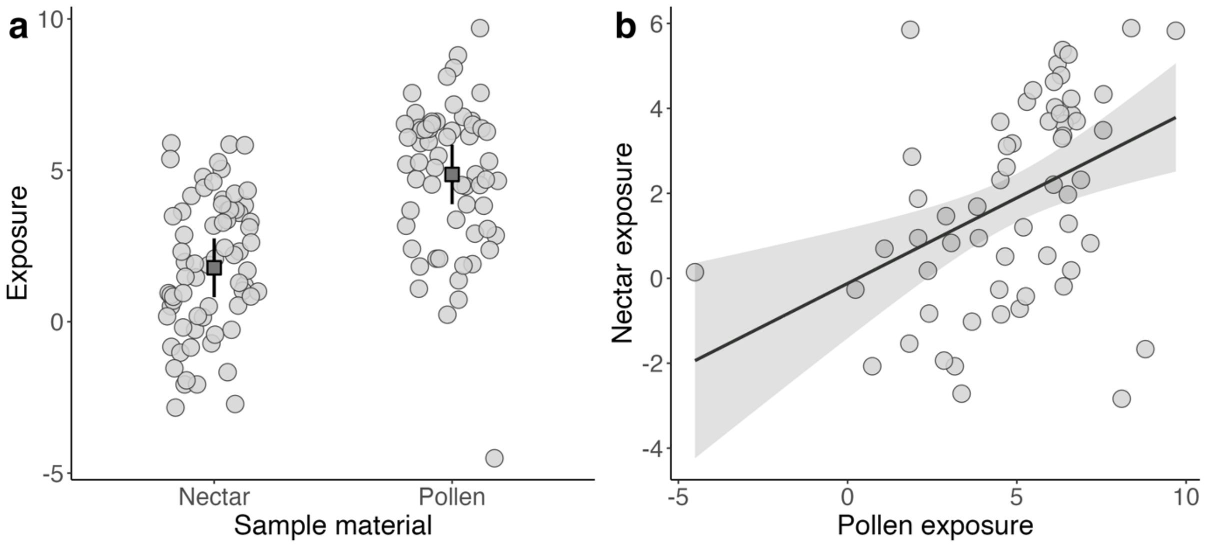

Figure S7. The level of exposure from pesticide residues was greater in pollen than in nectar (a), but the relative exposure correlated between sample materials. Black points and error bars (a) depict mean log transformed exposure and 95% confidence intervals. Predictions and 95% confidence intervals (a, b) are from linear mixed effects models with exposure log transformed.

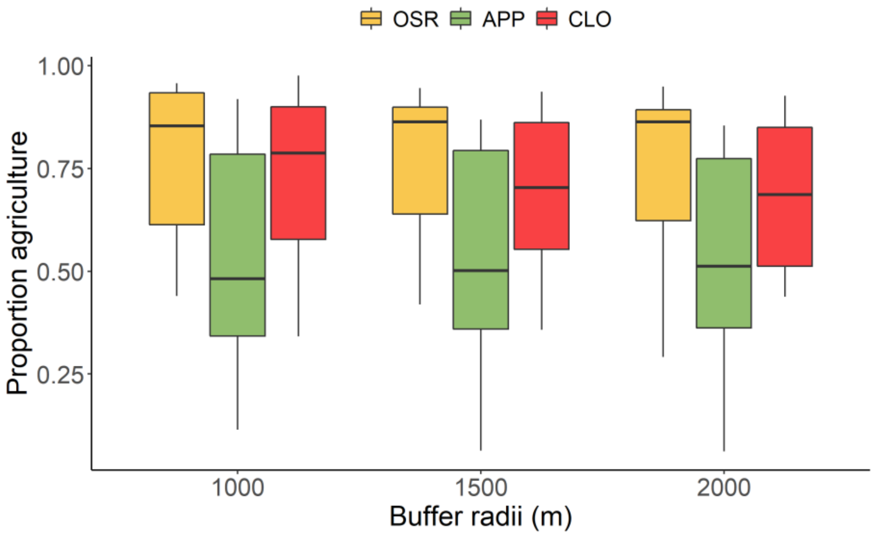

Figure S8. The proportion of agricultural land surrounding study sites was consistent across scales of buffer radii. Our three pollinator-dependent crops were oilseed rape (OSR), apple (APP), and red clover used for seed production (CLO).

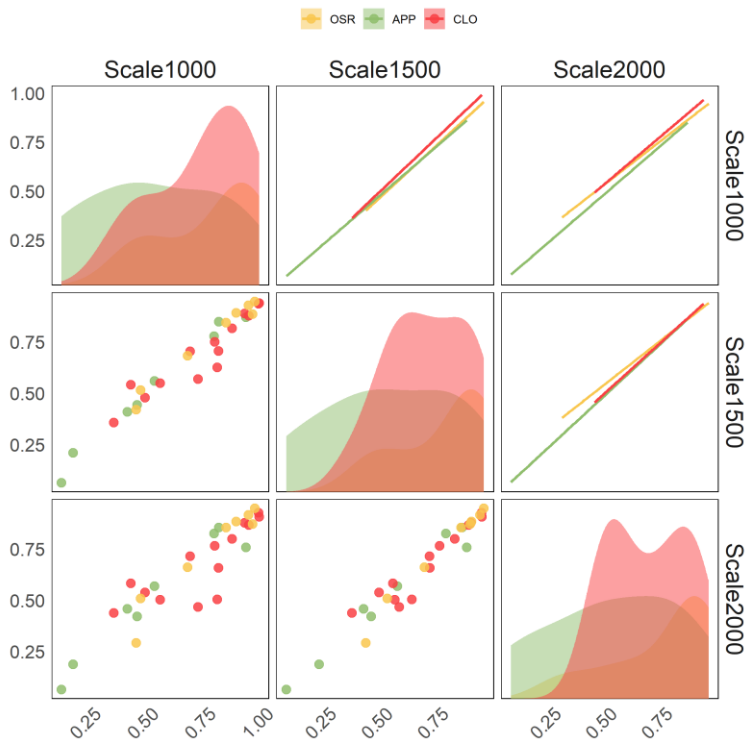

Figure S9. Correlation matrix of the proportion of agricultural land measured at three scales surrounding study sites centred on the three focal crops: oilseed rape (OSR), apple (APP), and red clover (CLO).

SUPPLEMENTARY TABLES

Table S1. Recommended chemical plant protection products (and their active ingredients) for use in the three focal cropping systems (OSR: oilseed rape; APP: apple; CLO: clover) in Sweden. Information on acaricides, fungicides, herbicides, and insecticides are from the Swedish Board of Agriculture in 2019, whilst herbicides in oilseed and clover are from the Swedish Board of Agriculture recommendations in 2022.

Type	Active ingredient(s)	Focal crop(s)	Product(s)
Acaricide	Fenpyroximate*	APP	Danitron 5 SC
Acaricide	Hexythiazox	APP	Nissorun SC
Fungicide	Azoxystrobin	OSR	Amistar, Mirador 250 EC, Quadris
Fungicide	Azoxystrobin + Difenoconazole	OSR	Amistar Gold
Fungicide	Azoxystrobin + Tebuconazole	OSR	Mirador Forte
Fungicide	Boscalid	OSR	Cantus
Fungicide	Boscalid + Pyraclostrobin	APP	Signum
Fungicide	Dithianon + Potassium phosphonates*	APP	Delan Pro
Fungicide	Dithianon*	APP	Delan WG
Fungicide	Dodine*	APP	Syllit 544 SC
Fungicide	Fenhexamide*	APP	Teldor WG 50
Fungicide	Kresoxime methyl*	APP	Candit
Fungicide	Metconazole + Mepiquat chloride	OSR	Caryx
Fungicide	Penconazole	APP	Topas 100 EC
Fungicide	Potassium bicarbonate*	APP	VitiSan
Fungicide	Prothioconazole	OSR	Proline EC
Fungicide	Prothioconazole + Fluopyram	OSR	Propulse SE 250
Fungicide	Prothioconazole + Tebuconazole	OSR; CLO	Folicur Xpert
Fungicide	Pyraclostrobin + Fluxapyroxad	OSR	Priaxor
Fungicide	Pyrimethanil*	APP	Scala
Fungicide	Sulphur*	APP	Kumulus DF
Fungicide	Thiophanate methyl*	APP	Topsin WG
Herbicide	Acetic acid*	APP	Ogräsättika
Herbicide	Clethodim	OSR; CLO	Select, Select Plus
Herbicide	Clomazone	OSR	Centium 36 CS, Kalif 360 CS
Herbicide	Clopyralid*	OSR	Cliophar 600 SL, Galera, Matrigon 72 SG
Herbicide	Cykloxidim	OSR; APP; CLO	Focus Ultra
Herbicide	Diquat dibromide salt*	APP	Diqua, Quad-Glob 200 SL, Reglone

Herbicide	Foramsulfuron + Iodosulfuronmethyl-sodium	APP	MaisTer
Herbicide	Geranium acid	APP	Finalsan Ogräs Effekt Proffs
Herbicide	Glyphosate	APP	Glyphosate based, multiple products
Herbicide	Halauxifen-methyl + Clopyralid	OSR	Korvetto
Herbicide	Halauxifen-methyl + Picloram	OSR	Belkar
Herbicide	Isoxaben*	APP	Gallery
Herbicide	MCPA*	CLO	Agroxone, Duplosan Max, Metaxon
Herbicide	Napropamide	OSR	Devrinol
Herbicide	Propaquizafop	OSR; APP; CLO	Agil100 EC, Zetrola
Herbicide	Propyzamide	OSR; APP; CLO	Kerb Flo 400
Herbicide	Quizalofop-P-ethyl*	OSR; CLO	Leopard, Targa Super 5SC
Herbicide	Tribenuron methyl*	CLO	Express 50 SX
Insecticide	Acetamiprid	OSR; APP	Mospilan SG
Insecticide	Alpha-cypermethrin	OSR; CLO	Fastac 50
Insecticide	Azadirachtin*	APP	NeemAzal-T/S
Insecticide	Beta-cyfluthrin	OSR; APP; CLO	Beta-Baythroid SC 025
Insecticide	Flonicamide*	APP	TEPPEKI
Insecticide	Indoxacarb	OSR; APP	Avaunt, Steward 30 WG
Insecticide	Paraffin oil*	APP	Fibro
Insecticide	Pymetrozine	OSR	Plenum, Plenum 50 WG
Insecticide	Rapeseed oil + Pyrethrins*	APP	Raptol
Insecticide	Spirotetramat*	APP	Movento SC 100
Insecticide	Tau-fluvalinate	OSR; CLO	Mavrik/Evure Neo
Insecticide	Thiacloprid	OSR; APP; CLO	Biscaya OD 240, Calypso SC 480

*compounds not screened in 2019; see Table S3.

Table S2. See separate excel sheet 'Table S2'.

Table S3. Differences in pesticide active ingredient composition based on PERMANOVA of Bray-Curtis dissimilarities between focal crops (OSR: oilseed rape; APP: apple; CLO: clover) (a) and bee species (b).

**(a) Focal crop comparisons**	**df**	**Sum of squares**	***F***	***P***
APP v. CLO	1.00	3.48	17.95	**< 0.001**
Residual	36.00	6.97		
Total	37.00	10.45		
APP v. OSR	1.00	0.86	4.27	**< 0.01**
Residual	44.00	8.87		
Total	45.00	9.73		
CLO v. OSR	1.00	2.98	15.65	**< 0.00**1
Residual	38.00	7.22		
Total	39.00	10.20		
**(b) Bee species comparisons**				
B. terrestris v. *A. mellifera*	1.00	0.47	1.75	0.11
Residual	44.00	11.77		
Total	45.00	12.24		
B. terrestris v. *O. bicornis*	1.00	0.51	1.89	0.07
Residual	36.00	9.78		
Total	37.00	10.29		
A. mellifera v. *O. bicornis*	1.00	0.86	3.85	**< 0.01**
Residual	38.00	8.46		
Total	39.00	9.31		

Table S4. Known pesticide applications at four oilseed rape (OSR), two apple (APP) and seven red clover (CLO) sites (table rows) where paired pollen and nectar samples were taken from returning *A. mellifera* and *B. terrestris* foragers 1-2, 4-6 and 12-16 days after application. At one apple and one oilseed rape site, farmers sprayed unknown fungicides.

Product(s)	Active ingredient(s)	Focal crop	Date applied
Mavrik/Evure Neo +	Tau-Fluvalinate	OSR	11/05/2019
Amistar	Azoxystrobin		
Steward 30 WG	Indoxacarb	APP	14/05/2019
Delan WG	Dithianon		16/05/2019
Fungicide(s)	-	OSR	14/05/2019
Biscaya OD 240 +	Thiacloprid	OSR	14/05/2019
Propulse SE 250	Fluopyram + Prothioconazole		
Mospilan SG +	Acetamiprid	OSR	15/05/2019
Mirador Forte	Azoxystrobin + Tebuconazole		
Fungicide(s)	-	APP	28/05/2019
Biscaya OD 240	Thiacloprid	CLO	14/06/2019
Biscaya OD 240	Thiacloprid	CLO	14/06/2019
Biscaya OD 240	Thiacloprid	CLO	17/06/2019
Mavrik	Tau-Fluvalinate	CLO	18/06/2019
Biscaya OD 240	Thiacloprid	CLO	19/06/2019
Biscaya OD 240	Thiacloprid	CLO	22/06/2019
Biscaya OD 240	Thiacloprid	CLO	23/06/2019
Biscaya OD 240	Thiacloprid	CLO	09/07/2019

Table S5. See separate excel sheet ' Table S5'.

Table S6. Key agricultural pollen groups were used to estimate the proportion of focal crop (bolded) or total agricultural pollen collected by the bee species (see Fig. 2d). Of all the screened pollen grains (n = 39 200), 13% belonged to the *Malus* group, 12% to the Brassicaceae group, 4% to the *Trifolium pratense* group, 1% in each of the *Trifolium repens* and *Solanum* spp. groups and <1% in the *Pisum sativum* and *Solanum* spp. groups. No pollen of *Vicia* spp. or *Helianthus annuus* was detected in the samples. Agricultural pollen groups were based on mass-flowering and bee-attractive Swedish crops, yet most pollen came from non-crop sources (Fig. 2d).

**Relevant Swedish crops**	**Group name**	**Description**
***Brassica napus* (oilseed rape)**, *Brassica rapa* (turnip rape)	Brassicaceae	all species of Brassicaceae (with pollen over 19 µm)
***Malus domesticus* (apple)**, *Pyrus communis* (pear), *Prunus avium* (cherry), *Prunus domestica* (plum), *Rubus idaeus* (raspberry)	*Malus*	all species of the following genera: *Malus, Prunus, Pyrus, Cotoneaster, Crataegus, Sorbus, Rubus, Amelanchier*
***Trifolium pratense* (red clover)**	*Trifolium pratense*	*Trifolium pratense* and *T. medium*
Trifolium repens (white clover), *Trifolium hybridum* (Alsike clover)	*Trifolium repens*	all species of *Trifolium* except those in the *T. pratense* group
Vicia faba (field beans)	*Vicia* spp.	all species of *Vicia*
Pisum sativum (peas)	*Pisum* sativum	only *P. sativum*
Solanum tuberosum (potato)	*Solanum* spp.	all species of *Solanum* except *S. dulcamara*
Helianthus annuus (sunflower)	*Helianthus annuus*	only *H. annuus*
Fragaria × ananassa (strawberry)	*Potentilla*	all species of *Potentilla* and *Fragaria*
