# Supplementary material for: Ecological traits interact with landscape context to determine bees’ pesticide risk
Source: Nat Ecol Evol. 2023 Feb 27;7(4):547–56. doi: 10.1038/s41559-023-01990-5 (PMC10089916; doi:10.1038/s41559-023-01990-5)
Supplement: Supplementary file 2 — Reporting Summary [file 41559_2023_1990_MOESM2_ESM.pdf]

nature portfolio

Corresponding author(s): Jessica Knapp

Last updated by author(s): Dec 17, 2022

Reporting Summary

Nature Portfolio wishes to improve the reproducibility of the work that we publish. This form provides structure for consistency and transparency in reporting. For further information on Nature Portfolio policies, see our Editorial Policies and the Editorial Policy Checklist.

Statistics

For all statistical analyses, confirm that the following items are present in the figure legend, table legend, main text, or Methods section.

n/a	Confirmed	
☐	☒	The exact sample size (*n*) for each experimental group/condition, given as a discrete number and unit of measurement
☐	☒	A statement on whether measurements were taken from distinct samples or whether the same sample was measured repeatedly
☐	☒	The statistical test(s) used AND whether they are one- or two-sided<br>*Only common tests should be described solely by name; describe more complex techniques in the Methods section.*
☐	☒	A description of all covariates tested
☐	☒	A description of any assumptions or corrections, such as tests of normality and adjustment for multiple comparisons
☐	☒	A full description of the statistical parameters including central tendency (e.g. means) or other basic estimates (e.g. regression coefficient) AND variation (e.g. standard deviation) or associated estimates of uncertainty (e.g. confidence intervals)
☐	☒	For null hypothesis testing, the test statistic (e.g. *F*, *t*, *r*) with confidence intervals, effect sizes, degrees of freedom and *P* value noted<br>*Give P values as exact values whenever suitable.*
☒	☐	For Bayesian analysis, information on the choice of priors and Markov chain Monte Carlo settings
☒	☐	For hierarchical and complex designs, identification of the appropriate level for tests and full reporting of outcomes
☒	☐	Estimates of effect sizes (e.g. Cohen's *d*, Pearson's *r*), indicating how they were calculated

Our web collection on statistics for biologists contains articles on many of the points above.

Software and code

Policy information about availability of computer code

Data collection	No software was used to collect data.
Data analysis	We used no custom computer code or algorithms for data analysis. All data were analysed in R version 4.1.1.

For manuscripts utilizing custom algorithms or software that are central to the research but not yet described in published literature, software must be made available to editors and reviewers. We strongly encourage code deposition in a community repository (e.g. GitHub). See the Nature Portfolio guidelines for submitting code & software for further information.

Data

Policy information about availability of data

All manuscripts must include a data availability statement. This statement should provide the following information, where applicable:

- Accession codes, unique identifiers, or web links for publicly available datasets
- A description of any restrictions on data availability
- For clinical datasets or third party data, please ensure that the statement adheres to our policy

The datasets generated and analysed during the current study are available from the corresponding author on reasonable request.

Field-specific reporting

Please select the one below that is the best fit for your research. If you are not sure, read the appropriate sections before making your selection.

☐ Life sciences ☐ Behavioural & social sciences ☒ Ecological, evolutionary & environmental sciences

For a reference copy of the document with all sections, see nature.com/documents/nr-reporting-summary-flat.pdf

Life sciences study design

All studies must disclose on these points even when the disclosure is negative.

Sample size	*Describe how sample size was determined, detailing any statistical methods used to predetermine sample size OR if no sample-size calculation was performed, describe how sample sizes were chosen and provide a rationale for why these sample sizes are sufficient.*
Data exclusions	*Describe any data exclusions. If no data were excluded from the analyses, state so OR if data were excluded, describe the exclusions and the rationale behind them, indicating whether exclusion criteria were pre-established.*
Replication	*Describe the measures taken to verify the reproducibility of the experimental findings. If all attempts at replication were successful, confirm this OR if there are any findings that were not replicated or cannot be reproduced, note this and describe why.*
Randomization	*Describe how samples/organisms/participants were allocated into experimental groups. If allocation was not random, describe how covariates were controlled OR if this is not relevant to your study, explain why.*
Blinding	*Describe whether the investigators were blinded to group allocation during data collection and/or analysis. If blinding was not possible, describe why OR explain why blinding was not relevant to your study.*

Behavioural & social sciences study design

All studies must disclose on these points even when the disclosure is negative.

Study description	*Briefly describe the study type including whether data are quantitative, qualitative, or mixed-methods (e.g. qualitative cross-sectional, quantitative experimental, mixed-methods case study).*
Research sample	*State the research sample (e.g. Harvard university undergraduates, villagers in rural India) and provide relevant demographic information (e.g. age, sex) and indicate whether the sample is representative. Provide a rationale for the study sample chosen. For studies involving existing datasets, please describe the dataset and source.*
Sampling strategy	*Describe the sampling procedure (e.g. random, snowball, stratified, convenience). Describe the statistical methods that were used to predetermine sample size OR if no sample-size calculation was performed, describe how sample sizes were chosen and provide a rationale for why these sample sizes are sufficient. For qualitative data, please indicate whether data saturation was considered, and what criteria were used to decide that no further sampling was needed.*
Data collection	*Provide details about the data collection procedure, including the instruments or devices used to record the data (e.g. pen and paper, computer, eye tracker, video or audio equipment) whether anyone was present besides the participant(s) and the researcher, and whether the researcher was blind to experimental condition and/or the study hypothesis during data collection.*
Timing	*Indicate the start and stop dates of data collection. If there is a gap between collection periods, state the dates for each sample cohort.*
Data exclusions	*If no data were excluded from the analyses, state so OR if data were excluded, provide the exact number of exclusions and the rationale behind them, indicating whether exclusion criteria were pre-established.*
Non-participation	*State how many participants dropped out/declined participation and the reason(s) given OR provide response rate OR state that no participants dropped out/declined participation.*
Randomization	*If participants were not allocated into experimental groups, state so OR describe how participants were allocated to groups, and if allocation was not random, describe how covariates were controlled.*

Ecological, evolutionary & environmental sciences study design

All studies must disclose on these points even when the disclosure is negative.

Study description	We selected 24 sites centred on three bee attractive flowering crops common in the region and flowering sequentially: oilseed rape (8 sites), apple (8 sites) and red clover (8 sites). We selected sites based on their surrounding proportion of agricultural land (2km radius) to ensure an even gradient (for each crop type) of agricultural land and, therefore, anticipated pesticide use.

Within the replicated sites, we sampled pollen from (1) Apis mellifera using pollen traps attached to two hives for 24 hours, (2) Bombus terrestris by capturing foragers (~20 across all six colonies) and euthanising them on dry ice as they returned to their colonies, and (3) multiple Osmia bicornis brood cells collected by females over the second half of the bloom period. We sampled pollen from A. mellifera and B. terrestris at two sampling intervals, coinciding with (1) the peak of crop bloom and (2) after crop bloom and for O. bicornis only at the end of crop bloom (evenly from all the available pollen). In total, we collected 48 samples (595 g) of A. mellifera-, 44 samples (11 g) of B. terrestris-, and 16 samples (70 g) of O. bicornis- collected pollen. During and after bloom samples were pooled for both A. mellifera and B. terrestris, resulting in 24 samples of A. mellifera-, 22 samples of B. terrestris- (all colonies died at two sites), and 16 samples of O. bicornis-collected pollen. We did not pool O. bicornis pollen over the bloom period since this species already combined pollen provisions on our behalf. In addition, we collected additional samples of corbicular pollen and and foraging bees for nectar extraction to produce paired pollen and nectar samples 1-2, 4-6 and 12-16 days after known pesticide applications at a selection of focal sites (n = 54 site and time point combinations for each material).

We conducted three primary analyses to understand agricultural pesticide risk to bee species. 1) Linear mixed models (LMMs) explore risk from pollen and use of agricultural pollen, with focal crop and bee species interacting with the proportion of agricultural land as fixed effects and site as a random intercept. 2) three linear models explore risk relationships among bee species' site-specific pollen collection, one for each species with the remaining bee species and focal crop as fixed effects. 3) LMMs to explore risk between sample materials, with sample material, focal crop and bee species as fixed effects, and sampling round nested in the site as a random effect.

Research sample

We conducted our study in Scania, Southern Sweden, an intensively farmed European production region. The selected sites cover multiple mass-flowering crops and agricultural contexts representing temperate agricultural landscapes and different pesticide use patterns. The selected three bee species exemplify different life-history traits that determine their activity. Both these aspects are important since bees are predicted to encounter pesticides as their activity intersects pesticide use patterns. The three bee species were A. mellifera, B. terrestris and O. bicornis, representing extensive, intermediate and limited bee foragers. A. mellifera colonies came from an experienced beekeeper, free of disease signs and managed according to good beekeeping practice by professional beekeepers. B. terrestris colonies were commercially reared (Biobest Biological Systems, Belgium), free of disease signs and were placed in large ventilated wooden boxes. O. bicornis cocoons came from a commercial breeder (Wildbiene & Partner, Switzerland), free of disease signs and provided unlimited nesting tubes.

Sampling strategy

No pre-study sample size calculation was performed. Instead, we relied on previous experience from the region on the needed replication for pesticide exposure and risk evaluations for bees. We have previously used a replication of 6-8 sites per factor level for bee pesticide exposure studies. In this study we used a replication of 8. We selected 24 sites in total centered on three bee attractive flowering crops common in the region and flowering sequentially: oilseed rape (8 sites), apple (8 sites) and red clover (8 sites). We selected sites based on their surrounding proportion of agricultural land (2km radius) to ensure a gradient (similar for each crop type) of agricultural land and, therefore, anticipated pesticide use, similarly to how we have selected sites in previous studies.

Within these sites, we collected a sufficient amount of pollen to represent pollen plant source and for pesticide residue quantification; we collected 48 samples covering the 24 sites and 2 time points (in total 1210 g) from A. mellifera and 44 samples covering 22 sites (all colonies were lost at two apple sites which excluded sampling) and 2 time points (in total 1309 pollen loads) from B. terrestris. Sampling was restricted beyond this to exclude negatively affecting colony functioning. Furthermore, we collected all pollen possible from O. bicornis, which were 16 samples (71 g).

Data collection

The authors and paid research assistants collected the data and samples of bees and bee-collected pollen from April to August 2019, using pollen traps and insect nets. Sample per bee species, type of material, site and sampling time point were separately stored in tubes on ice until return to the laboratory at the end of the day, after which samples were frozen (-20C). Pollen samples and bee bodies were sent to the analytical laboratory for nectar extraction and pesticide residue analysis. Half of the sample pollen was used to identify plant species origin. Data were noted in spreadsheets on sample origin and amount of material along with pesticide identity and concentration, as well as plant species origin for pollen samples. Land use data was based on the IACS Spatial Data Layer provided by the Swedish Board of Agriculture for the study year (2019) and extracted using R. Data on pesticide properties and toxicity information were extracted from the Pesticide Properties Database (PPDB) hosted by the University of Hertfordshire.

Timing and spatial scale

We collected samples of pollen and bees from each focal crop field during and after bloom. Our crops bloomed sequentially: oilseed rape from April-May, apple from May-June and red clover from June-August, so that in addition to covering focal crop blooms we also covered most of the relevant season for the focal bee species. Samples at oilseed rape sites were collected 14-24 May (mid-bloom) and 22 May-7 June (end-post bloom), samples at apple sites were collected 20 May-2 June (mid-bloom) and 21 May-11 June (end-post bloom) and samples at clover sites were collected 25 June-18 July (mid-bloom) and 12-26 July (end bloom). In addition, we collected additional samples coinciding with a known pesticide application (before or during flowering) at a selection of focal sites during 14 May-25 July, all in 2019. Thus all samples were collected from May to July 2019. We conducted our study across Scania, Southern Sweden, an intensively farmed European production region covering about 100 x 100 km.

Data exclusions

No data were excluded.

Reproducibility

All data will be made freely available upon publication, and our manuscript describes our methodology in full. We have made no attempts to repeat this experiment.

Randomization

Sites centered on the three focal crops were selected to cover similar gradients of agricultural land and, therefore, anticipated pesticide use in the landscape. Proportion agricultural land was checked for consistency across bee relevant scales (1000, 1500, 2000 m radius). We matched honeybee hives and bumblebee colonies by their strength and randomly allocated them and Osmia cocoons to sites to ensure similar foraging efforts.

Blinding

Blinding during field sampling was not possible since focal bee species, focal crop and landscape context were clearly visible.

Did the study involve field work? ☒ Yes ☐ No

Field work, collection and transport

Field conditions	We collected all data on warm and dry days when bees were active.
Location	Scania, Southern Sweden: 55.9903° N, 13.5958° E
Access & import/export	B. terrestris colonies were imported through a company (Lindesro AB) that held an import permit according to the Swedish Board of Agriculture. A. mellifera colonies were assessed for their health and moved to the study sites according to approval by the County Administrative Board. We needed no other permits for this study, and all farmers provided their full permission for us to access their land for fieldwork.
Disturbance	Disturbance was limited by removing the managed bees after the sampling was concluded.

Reporting for specific materials, systems and methods

We require information from authors about some types of materials, experimental systems and methods used in many studies. Here, indicate whether each material, system or method listed is relevant to your study. If you are not sure if a list item applies to your research, read the appropriate section before selecting a response.

Materials & experimental systems

n/a	Involved in the study	
☒	☐	Antibodies
☒	☐	Eukaryotic cell lines
☒	☐	Palaeontology and archaeology
☐	☒	Animals and other organisms
☒	☐	Human research participants
☒	☐	Clinical data
☒	☐	Dual use research of concern

Methods

n/a	Involved in the study	
☒	☐	ChIP-seq
☒	☐	Flow cytometry
☒	☐	MRI-based neuroimaging

Antibodies

Antibodies used	*Describe all antibodies used in the study; as applicable, provide supplier name, catalog number, clone name, and lot number.*
Validation	*Describe the validation of each primary antibody for the species and application, noting any validation statements on the manufacturer's website, relevant citations, antibody profiles in online databases, or data provided in the manuscript.*

Eukaryotic cell lines

Policy information about cell lines

Cell line source(s)	*State the source of each cell line used.*
Authentication	*Describe the authentication procedures for each cell line used OR declare that none of the cell lines used were authenticated.*
Mycoplasma contamination	*Confirm that all cell lines tested negative for mycoplasma contamination OR describe the results of the testing for mycoplasma contamination OR declare that the cell lines were not tested for mycoplasma contamination.*
Commonly misidentified lines (See ICLAC register)	*Name any commonly misidentified cell lines used in the study and provide a rationale for their use.*

Palaeontology and Archaeology

Specimen provenance	*Provide provenance information for specimens and describe permits that were obtained for the work (including the name of the issuing authority, the date of issue, and any identifying information). Permits should encompass collection and, where applicable, export.*
Specimen deposition	*Indicate where the specimens have been deposited to permit free access by other researchers.*
Dating methods	*If new dates are provided, describe how they were obtained (e.g. collection, storage, sample pretreatment and measurement), where they were obtained (i.e. lab name), the calibration program and the protocol for quality assurance OR state that no new dates are provided.*

☐ Tick this box to confirm that the raw and calibrated dates are available in the paper or in Supplementary Information.

Ethics oversight	*Identify the organization(s) that approved or provided guidance on the study protocol, OR state that no ethical approval or guidance*

Ethics oversight | *was required and explain why not.*

Note that full information on the approval of the study protocol must also be provided in the manuscript.

Animals and other organisms

Policy information about studies involving animals; ARRIVE guidelines recommended for reporting animal research

Laboratory animals	We collected samples from three managed bee species at focal sites: Apis mellifera, Bombus terrestris, and Osmia bicornis.
Wild animals	Our study did not involve wild animals.
Field-collected samples	We stored pollen and bee samples at -20C before screening for pesticide compounds included in the Swedish national monitoring scheme, following established protocols at the analytical laboratory.
Ethics oversight	No ethical approval is required for insect collections in Sweden.

Note that full information on the approval of the study protocol must also be provided in the manuscript.

Human research participants

Policy information about studies involving human research participants

Population characteristics	*Describe the covariate-relevant population characteristics of the human research participants (e.g. age, gender, genotypic information, past and current diagnosis and treatment categories). If you filled out the behavioural & social sciences study design questions and have nothing to add here, write "See above."*
Recruitment	*Describe how participants were recruited. Outline any potential self-selection bias or other biases that may be present and how these are likely to impact results.*
Ethics oversight	*Identify the organization(s) that approved the study protocol.*

Note that full information on the approval of the study protocol must also be provided in the manuscript.

Clinical data

Policy information about clinical studies

All manuscripts should comply with the ICMJE guidelines for publication of clinical research and a completed CONSORT checklist must be included with all submissions.

Clinical trial registration	*Provide the trial registration number from ClinicalTrials.gov or an equivalent agency.*
Study protocol	*Note where the full trial protocol can be accessed OR if not available, explain why.*
Data collection	*Describe the settings and locales of data collection, noting the time periods of recruitment and data collection.*
Outcomes	*Describe how you pre-defined primary and secondary outcome measures and how you assessed these measures.*

Dual use research of concern

Policy information about dual use research of concern

Hazards

Could the accidental, deliberate or reckless misuse of agents or technologies generated in the work, or the application of information presented in the manuscript, pose a threat to:

No	Yes	
☐	☐	Public health
☐	☐	National security
☐	☐	Crops and/or livestock
☐	☐	Ecosystems
☐	☐	Any other significant area

Experiments of concern

Does the work involve any of these experiments of concern:

No	Yes	
☐	☐	Demonstrate how to render a vaccine ineffective
☐	☐	Confer resistance to therapeutically useful antibiotics or antiviral agents
☐	☐	Enhance the virulence of a pathogen or render a nonpathogen virulent
☐	☐	Increase transmissibility of a pathogen
☐	☐	Alter the host range of a pathogen
☐	☐	Enable evasion of diagnostic/detection modalities
☐	☐	Enable the weaponization of a biological agent or toxin
☐	☐	Any other potentially harmful combination of experiments and agents

ChIP-seq

Data deposition

☐ Confirm that both raw and final processed data have been deposited in a public database such as GEO.

☐ Confirm that you have deposited or provided access to graph files (e.g. BED files) for the called peaks.

Data access links<br>*May remain private before publication.*	*For "Initial submission" or "Revised version" documents, provide reviewer access links. For your "Final submission" document, provide a link to the deposited data.*
Files in database submission	*Provide a list of all files available in the database submission.*
Genome browser session<br>(e.g. UCSC)	*Provide a link to an anonymized genome browser session for "Initial submission" and "Revised version" documents only, to enable peer review. Write "no longer applicable" for "Final submission" documents.*

Methodology

Replicates	*Describe the experimental replicates, specifying number, type and replicate agreement.*
Sequencing depth	*Describe the sequencing depth for each experiment, providing the total number of reads, uniquely mapped reads, length of reads and whether they were paired- or single-end.*
Antibodies	*Describe the antibodies used for the ChIP-seq experiments; as applicable, provide supplier name, catalog number, clone name, and lot number.*
Peak calling parameters	*Specify the command line program and parameters used for read mapping and peak calling, including the ChIP, control and index files used.*
Data quality	*Describe the methods used to ensure data quality in full detail, including how many peaks are at FDR 5% and above 5-fold enrichment.*
Software	*Describe the software used to collect and analyze the ChIP-seq data. For custom code that has been deposited into a community repository, provide accession details.*

Flow Cytometry

Plots

Confirm that:

☐ The axis labels state the marker and fluorochrome used (e.g. CD4-FITC).

☐ The axis scales are clearly visible. Include numbers along axes only for bottom left plot of group (a 'group' is an analysis of identical markers).

☐ All plots are contour plots with outliers or pseudocolor plots.

☐ A numerical value for number of cells or percentage (with statistics) is provided.

Methodology

Sample preparation	*Describe the sample preparation, detailing the biological source of the cells and any tissue processing steps used.*
Instrument	*Identify the instrument used for data collection, specifying make and model number.*

Software | *Describe the software used to collect and analyze the flow cytometry data. For custom code that has been deposited into a community repository, provide accession details.*

Cell population abundance | *Describe the abundance of the relevant cell populations within post-sort fractions, providing details on the purity of the samples and how it was determined.*

Gating strategy | *Describe the gating strategy used for all relevant experiments, specifying the preliminary FSC/SSC gates of the starting cell population, indicating where boundaries between "positive" and "negative" staining cell populations are defined.*

☐ Tick this box to confirm that a figure exemplifying the gating strategy is provided in the Supplementary Information.

Magnetic resonance imaging

Experimental design

Design type | *Indicate task or resting state; event-related or block design.*

Design specifications | *Specify the number of blocks, trials or experimental units per session and/or subject, and specify the length of each trial or block (if trials are blocked) and interval between trials.*

Behavioral performance measures | *State number and/or type of variables recorded (e.g. correct button press, response time) and what statistics were used to establish that the subjects were performing the task as expected (e.g. mean, range, and/or standard deviation across subjects).*

Acquisition

Imaging type(s) | *Specify: functional, structural, diffusion, perfusion.*

Field strength | *Specify in Tesla*

Sequence & imaging parameters | *Specify the pulse sequence type (gradient echo, spin echo, etc.), imaging type (EPI, spiral, etc.), field of view, matrix size, slice thickness, orientation and TE/TR/flip angle.*

Area of acquisition | *State whether a whole brain scan was used OR define the area of acquisition, describing how the region was determined.*

Diffusion MRI ☐ Used ☐ Not used

Preprocessing

Preprocessing software | *Provide detail on software version and revision number and on specific parameters (model/functions, brain extraction, segmentation, smoothing kernel size, etc.).*

Normalization | *If data were normalized/standardized, describe the approach(es): specify linear or non-linear and define image types used for transformation OR indicate that data were not normalized and explain rationale for lack of normalization.*

Normalization template | *Describe the template used for normalization/transformation, specifying subject space or group standardized space (e.g. original Talairach, MNI305, ICBM152) OR indicate that the data were not normalized.*

Noise and artifact removal | *Describe your procedure(s) for artifact and structured noise removal, specifying motion parameters, tissue signals and physiological signals (heart rate, respiration).*

Volume censoring | *Define your software and/or method and criteria for volume censoring, and state the extent of such censoring.*

Statistical modeling & inference

Model type and settings | *Specify type (mass univariate, multivariate, RSA, predictive, etc.) and describe essential details of the model at the first and second levels (e.g. fixed, random or mixed effects; drift or auto-correlation).*

Effect(s) tested | *Define precise effect in terms of the task or stimulus conditions instead of psychological concepts and indicate whether ANOVA or factorial designs were used.*

Specify type of analysis: ☐ Whole brain ☐ ROI-based ☐ Both

Statistic type for inference (See Eklund et al. 2016) | *Specify voxel-wise or cluster-wise and report all relevant parameters for cluster-wise methods.*

Correction | *Describe the type of correction and how it is obtained for multiple comparisons (e.g. FWE, FDR, permutation or Monte Carlo).*

Models & analysis

n/a	Involved in the study
☐	☐ Functional and/or effective connectivity
☐	☐ Graph analysis
☐	☐ Multivariate modeling or predictive analysis

Functional and/or effective connectivity	*Report the measures of dependence used and the model details (e.g. Pearson correlation, partial correlation, mutual information).*
Graph analysis	*Report the dependent variable and connectivity measure, specifying weighted graph or binarized graph, subject- or group-level, and the global and/or node summaries used (e.g. clustering coefficient, efficiency, etc.).*
Multivariate modeling and predictive analysis	*Specify independent variables, features extraction and dimension reduction, model, training and evaluation metrics.*
